# Introducing a Dengue Vaccine to Mexico: Development of a System for Evidence-Based Public Policy Recommendations

**DOI:** 10.1371/journal.pntd.0003009

**Published:** 2014-07-31

**Authors:** Miguel Betancourt-Cravioto, Pablo Kuri-Morales, Jesús Felipe González-Roldán, Roberto Tapia-Conyer, the Mexican Dengue Expert Group

**Affiliations:** 1 Carlos Slim Health Institute, Mexico City, Mexico; 2 Ministry of Health, Mexico City, Mexico; 3 National Center for Disease Control and Prevention, Ministry of Health, Mexico City, Mexico; 4 Carlos Slim Foundation, Mexico City, Mexico; Pediatric Dengue Vaccine Initiative, United States of America

## Introduction

The incidence of dengue fever (DF) is steadily increasing in Mexico [1], [2], as it is in the rest of Latin America [3], burdening health systems with the consequent morbidity and mortality [4]–[6]. The incidence of DF in Mexico has increased from 1.7 cases per 100,000 inhabitants in 2000 to 43.03 cases per 100,000 inhabitants in 2012 (Table 1) [1], [2]. Growing urbanization, human migration, climate change, and ecological disruption have facilitated the expansion of dengue's vectors, *Aedes aegypti* and *A. albopictus* [3], [4]. This increase, which could be solely attributed to an improvement in epidemiological surveillance, in reality has a much more complex explanation. According to reports from the Mexican Secretariat of Health, DF in Mexico follows a cyclical pattern of approximately five years per cycle, characterized by a sudden and dramatic rise in cases subsequently followed by a period of decrement that ultimately results in years with a low number of cases. This pattern seems to have its origin in the introduction/reintroduction of new serotypes among the population [2].

**Table 1:** Evolution of the incidence of dengue fever cases in Mexico between 2000 and 2012.

Year	Incidence per 100,000 Inhabitants
**2000 ** [1]	**1.70**
**2005 ** [1]	**16.40**
**2012 ** [2]	**43.03**

Globally, an estimated 2,500,000,000 to 3,000,000,000 individuals are at risk of infection. Each year 50 million people fall ill, and 20,000 people die as a result of dengue [5]–[7]. These trends will continue to worsen in the foreseeable future [3], [7].

Even at its current level, dengue overloads healthcare systems, particularly in developing countries in which resources are scarce [4]. The direct and indirect costs of dengue are high and impose a considerable financial burden on those affected [2], [8]. The most commonly used dengue control measure—vector control programs—have had poor effects on dengue incidence and are difficult to implement in a sustainable fashion [9]–[11]. Also, these programs are costly and have limited effect because of the difficulty of destroying all mosquitoes in an area [9]. Additionally, there are no effective antivirals available to treat the disease. However, as a result of much progress in research and development over the last decade [12], there is a prospect of a safe and effective preventive vaccine becoming available soon [13]–[16].

In 2012, Sanofi-Pasteur (SP) published the results of a Phase IIB clinical trial carried out in Thailand to test the efficacy of its recombinant, live-attenuated, tetravalent dengue vaccine. The authors reported that although the vaccine showed less efficacy than projected, this was not significant, and the vaccine resulted immunogenic for the four dengue serotypes and protected against serotypes 1, 3, and 4 [17]. Irregardless of the results, the authors of the present report consider that this exercise has been successful and a step forward in the process towards obtaining a much-needed dengue vaccine. Nevertheless, it will be important to assess further data, particularly from the ongoing Phase III studies in Latin America and Asia, in which Mexico is participating with several clinical sites [18].

A dengue vaccine would change the paradigm of dengue control by providing invaluable support to currently available prevention and control measures. Importantly, as a disease most prevalent in developing regions, a dengue vaccine would be primarily aimed at low- and middle-income countries (LMICs) [2], [16], a reversal of the traditional situation. Historically, LMICs wait for vaccines to become available and licensed in developed countries before adopting it themselves once an evidence base is generated and prices have come down to affordable levels [19], [20].

The prospect of a dengue vaccine presents LMICs, like Mexico, with an opportunity to strengthen their decision-making capacity in order to make timely and well-informed decisions about the introduction of new interventions for disease prevention and control. Improving the decision-making process will help LMICs become early adopters of public health interventions [21] and help close the time gap between innovation and access to new vaccines in such countries [22].

For early access to the dengue vaccine, countries will need to be proactive, since early access entails the timely development of adoption plans with active involvement of all sectors of society, including academic institutions, nonprofit organizations, ministries of health and finance, and health service providers [21]. Consequently, LMICs will need to prepare early and engage with all stakeholders to accelerate the process of making the vaccine available to their populations.

## The Mexican Dengue Expert Group

In anticipation of this changing landscape, the Mexican Federal Ministry of Health (FMoH), in partnership with the Carlos Slim Health Institute, undertook the development of a national strategy for the introduction of a dengue vaccine. This exercise aimed to establish evidence-based policy recommendations to enable the early adoption of a dengue vaccine in Mexico incorporating evidence-based innovative strategies and approaches. The resulting recommendations were presented to national public health authorities for their use in the decision-making process. The recommendations will be shared with other countries, with a goal of developing a regional strategy for the introduction and use of dengue vaccines.

**Figure 1:**
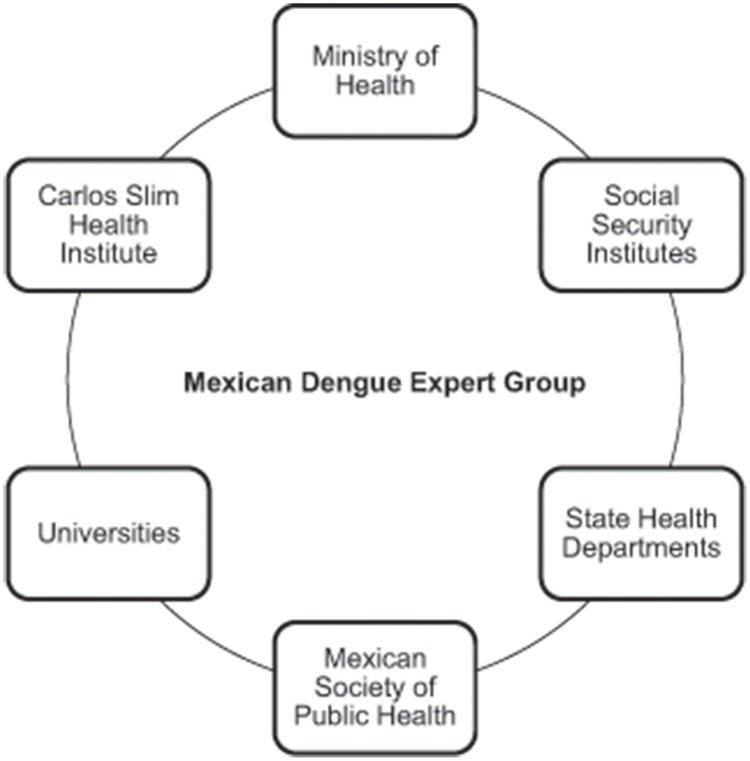
Composition of the MDEG.

To achieve these objectives, the Mexican Dengue Expert Group (MDEG) (Figure 1) was formed as an ad hoc, independent, multidisciplinary think tank, integrating leading scientists, researchers, federal and state public health officials, decision makers from academic institutions, public health providers, public health institutions, civil society, and the private sector. The MDEG was tasked with evaluating, in a six-month period, the value of a novel dengue vaccine and how such a vaccine could be best introduced.

The MDEG's work, conducted between May and October 2012, was organized into five lines of action, each identified as important to address for the early adoption of a dengue vaccine. A multidisciplinary working group (WG) of relevant experts was established for each area (Table 2). Communication between groups and with vaccine producers in Mexico was encouraged and driven by the goals and objectives set for each WG at the plenary sessions of the MDEG. The information shared by vaccine producers provided important elements to incorporate in discussions, such as possible time frames for vaccine rollout, probable vaccine schemes, and information about the progress of each vaccine under development. The MDEG integrated the results of its general and WG discussions into policy recommendations for the introduction of a dengue vaccine into the national immunization schedule. A document with the policy recommendations was presented in late 2012 to the FMoH for consideration. Although it was not meant to be a legally binding document, because of the relevance of the experts involved and the institutions represented in the MDEG, the recommendations have become a reference and guide for policy making and program implementation in order to prepare the country for the introduction of the dengue vaccine in Mexico in the short term.

**Table 2:** The MDEG constituent multidisciplinary working groups.

MDEG: Constituent Multidisciplinary Working Groups	
**1**	Epidemiologic Information Systems, Burden of Disease, and Impact Modeling
**2**	Economic Analysis and Financial Mechanisms
**3**	Immunizations Systems
**4**	Regulatory Issues
**5**	Social Communication and Health Promotion

## Results from the MDEG Working Groups

### 1. Epidemiologic Information Systems, Burden of Disease, and Impact Modeling

After reviewing and analyzing available information on the burden of dengue in Mexico, surveillance systems and networks, diagnostic protocols, and epidemiologic modeling, the WG proposed the following recommendations:

- Strengthen the supervision of the national dengue surveillance system with periodic underreporting analyses to ensure proper reporting of cases.
- Actively utilize epidemiologic data to better stratify risk areas, identify regions that would benefit most from the vaccine, and evaluate its effectiveness. Specifically, use the data to define geographical areas for vaccination considering the incidence of the disease, circulating viruses, and age groups affected.
- Maintain the use of the International Classification of Diseases-10 [23] DF and dengue hemorrhagic fever definitions for epidemiologic surveillance in order to ensure the comparability of data collected before and after vaccine introduction. The revised World Health Organization classification, having been created to facilitate timely and adequate therapeutic decision making, should continue to be used in clinical settings [24]. However, the relevance of this revised classification for epidemiological surveillance still needs to be assessed.
- Strengthen virological surveillance with genetic, phylogenetic, and phylogeographic studies pre– and post–vaccine introduction.
- Carry out routine seroprevalence surveys to help define geographic areas and age groups for vaccine introduction.
- Develop mathematic models of the impact of vaccine introduction in Mexico to provide decision makers with data on possible scenarios before, during, and after vaccine deployment.

### 2. Economic Analysis and Financial Mechanisms

This WG created different costing scenarios for the introduction of the vaccine, taking into account other existing public health priorities and currently available dengue prevention and control strategies. The group also looked into cost-effectiveness and cost-benefit analyses and defined costing scenarios and possible financing schemes stressing affordability and sustainability.

Based on available evidence, the group concluded that evaluations of the economic burden of dengue in Mexico are insufficient to support decision-making. Existing evaluations were deemed unsuitable for comparison because of the use of different methodologies. Consequently, the group proposed the following actions:

- Establish a common methodology for economic burden studies.
- Carry out studies to ascertain the real economic burden of dengue in Mexico.
- Use data on economic burden for cost-effectiveness studies comparing different dengue prevention and control strategies with the vaccine, either as the only intervention or in conjunction with the rest of the strategies (vector control, health promotion, disease prevention, and early treatment).

The acquisition of this information will be essential in the coming years for establishing the financing sources and mechanisms for the introduction of the vaccine. Currently in Mexico, most of the vaccines purchased are paid for by the federal government and distributed for free through the public health sector. The private immunization market accounts for less than 1% of the total. It is expected that most of the dengue vaccine would be similarly purchased by the government and distributed free of charge to the population. A smaller amount will surely be available for a fee through the private sector [25], [26].

Funding for the National Immunizations Program in Mexico is negotiated on a yearly basis within the budget of the FMoH, based on institutional needs and population estimates. Customarily, the allocated budget for vaccines of the previous year is the minimum to be allocated in the following fiscal period, and it cannot go below that figure.

In the case of the introduction of a new vaccine such as one for dengue, once the additional budget for the purchase and distribution of the immunizations is approved by the Ministry of Finance, it will not be revoked [27].

The negotiation for the pricing of vaccines is carried out directly with manufacturers by the FMoH, through the National Center for the Health of Children and Adolescents, in representation of all the institutions in the public health sector. Vaccine prices in the private sector are driven by market forces [26].

### 3. Immunizations Systems

The introduction of new vaccines poses a great burden on existing infrastructure for vaccine delivery. The Immunizations Systems WG carried out a situation analysis of the Mexican National Immunization Program (MNIP), including cold-chain capacity, logistics, vaccination-coverage information systems, and personnel capacity. Elements considered included age groups to immunize; phased, incremental, or universal introduction; geographic areas; and the need for catch-up campaigns. A final element was to define the expected interaction between the MNIP and the National Vector Control Program.

The situation analysis found sufficiently available surge capacity, both in terms of human resources and infrastructure, to support the introduction of a new vaccine. Also, in the past few years, the Mexican FMoH has invested important resources to expand cold-chain capacity at all levels. This analysis will have to be adjusted once the final presentation of the vaccine(s) is known, results from conclusive burden of disease and econometric studies become available, and the country's needs are definitely established.

However, based on the available epidemiologic surveillance data and current information from manufacturers, the Immunizations Systems WG proposed the following recommendations:

**Table 3:** Proposed vaccination scheme by the Immunizations Systems Working Group.

Vaccination Scheme Proposed by the Immunizations Systems Working Group*			
**Years after introduction**	**Cohorts (years of age)**	**Population to be vaccinated (per year)** **	**Required doses (million)** **
**First year**	2, 3, and 4	5 to 8 million	15 to 24
**Second year**	2, 6, and 7	5 to 8 million	15 to 24
**Third year onwards**	2	2 to 3 million	6 to 9

*Considers three doses per child as per the recommended schedule [28].

** The annual birth cohort is estimated at 2 million children.

I. To establish baseline measurements to evaluate the impact of the vaccination strategy, strengthen pre-introduction surveillance through the creation of epidemiologic catchment areas. This strategy is in line with proposed improvements to dengue epidemiological surveillance by the FMoH in Mexico that will begin to be implemented in the next few years.
II. The vaccination strategy would initially cover children from endemic areas with high dengue transmission rates. In later years the program would be expanded to areas with lower transmission rates. The availability of vaccine from manufacturers and of resources for vaccine purchase and distribution will guide this process.
III. Begin vaccination at two years of age and carry out a catch-up strategy for children up to four years of age over the course of two years (Table 3). This would mean vaccinating a population of 5 to 8 million children between two and seven years of age per year during the first two years and 2 to 3 million children annually from the third year on.
  The rationale behind this recommendation is the idea of vaccinating the youngest age group at risk of the disease in order to maximize the benefit of the protection provided by the vaccine [28]. The most recent epidemiological data shows that the highest dengue fever incidence in Mexico occurs between ten and 19 years of age. With the proposed strategy, in three years' time, the two- to six-year-old age group would be covered, significantly reducing the number of susceptible individuals [2].
IV. Vaccinate through dedicated campaigns coinciding with National Health Weeks (NHWs), which will facilitate vaccine delivery to the population.

Every year three NHWs take place in Mexico (February, May, and October). Considering the currently proposed vaccination schedule of 0, 6, and 12 months, vaccination campaigns could take place during the second and third NHWs [29].

V. Adequately train vaccination teams on the novel dengue vaccine.
VI. Align the dengue vaccination program with the national dengue control program.
VII. Develop personnel training programs on specific aspects of managing and administering the new vaccine to ensure the adequate impact of the program and to reduce vaccine-related accidents.
VIII. Evaluate final vaccine effectiveness and safety results. Since there is still a lack of data on the coadministration of the dengue vaccine and other immunizations, the correct implementation of postmarketing surveillance will be essential to collect data on the safety of the vaccine.
IX. Postmarketing pharmacovigilance will be carried out through the National Pharmacovigilance System [30]. It was recommended by the Immunizations Systems WG that the Federal Commission for the Protection against Health Risks (COFEPRIS) review and adjust its procedures and bylaws to ensure that the pharmacovigilance system is sensitive enough for the possible adverse consequences of the introduction of the new immunization.

### 4. Regulatory (COFEPRIS)

The evaluation of regulatory and licensing issues for vaccine introduction was led by the Mexican National Regulatory Agency (NRA): COFEPRIS.

Although the regulatory work for the introduction of the vaccine will mostly take place once manufacturers begin the registration process, the group recommended several preparatory steps that will allow for timely evaluation of safety and efficacy data to support decision-making once the licensing procedure starts:

- Continuously review industry data, particularly results from Phase II and III studies made available by laboratories, including efficacy reports and safety profiles as well as reports on public health impact and cost and availability estimations.
- Continuously update and streamline procedures for vaccine licensing and registration to facilitate the introduction of new vaccines.
- In light of the complex dengue vaccine environment with several vaccine candidates under development, analyze the possibility and consequences of licensing several vaccines at the same time.
- Strengthen postmarketing surveillance systems in collaboration with public health institutions and pharmaceutical companies as established in the National Bylaw for Pharmacovigilance [30]. This synergy will help strengthen the existing pharmacosurveillance procedures [31].

### 5. Social Communication and Health Promotion

The objective of this WG was to develop strategies to activate community participation for the introduction of the new vaccine. The group acknowledged the need to inform and create public awareness about the vaccine before and during introduction. The group's recommendations focused on the generation of messages to promote vaccine adoption by the community and the utilization of social marketing to disseminate information about the introduction of the new vaccine to the public.

It was recommended that the following elements be taken into account when developing the social communication strategy for the dengue vaccination program:

- General information on the vaccine, including issues such as safety and efficacy
- Explanations on why only certain regions and age groups are included in the vaccination strategy
- Emphasis on the need for an integrated approach to dengue control; vaccines will not be the sole solution, but an important addition to strategies already in place

## Discussion

The exercise carried out by the MDEG represents the first step towards enabling early adoption of a dengue vaccine in Mexico and other LMICs. The development of this evidence-based proactive strategy represents a move from the traditional paradigm of waiting for evidence to be generated in other countries before making a local decision. Instead, this strategy proposes that LMICs actively participate in the generation of evidence, the analysis of data from clinical trials, and the estimation of the burden of disease. The MDEG's strategy also promotes the use of national-level evidence by decision makers to create a sustainable immunization program with all the resources required for the adoption of the new vaccine.

While similar initiatives to strengthen evidence-based national decision-making for the introduction of new vaccines have been developed, these have focused on existing vaccines for diseases such as *Haemophilus influenzae* type B and pneumococcal infections [32]. However, Mexico has already had experience in being an early introducer of vaccines, such as in the case of the 2005 rotavirus vaccine, in which it played a crucial role in generating the appropriate evidence for the first licensing of the vaccine worldwide, doing so even before the United States Food and Drug Administration (FDA) or the European Medicines Agency (EMEA) [33]. This action by the Mexican government allowed for the vaccine to be included immediately in the national vaccination schedule and not have to wait for years for FDA or EMEA licensure. In turn, it has been estimated that the new vaccine has saved over 1,000 lives yearly in Mexico since its introduction [34].

It has been argued that NRAs in developing countries are less rigorous than those in higher income countries, suggesting that there could be risks to the population due to inadequate regulation practices. Nevertheless, LMICs have been strengthening their regulatory authorities [35]. In fact, in 2012 COFEPRIS received certification by the Pan American Health Organization as a Regional Reference Regulatory Agency for Drugs and Vaccines [36].

In carrying out the exercise described here, the Mexican National Health System is preparing in advance to introduce imminent dengue vaccines.

In summary, the MDEG produced the following recommendations:

- Strengthen epidemiologic, entomologic, and virological surveillance and obtain data necessary for impact modelling of dengue vaccine introduction.
- Carry out studies of the economic burden of dengue in Mexico necessary for cost-benefit and cost-effectiveness analyses to support financial decisions.
- Introduce the vaccine as soon as it becomes available.
- Invite potential vaccine producers to engage with the national regulatory authority to facilitate registration and licensing processes.
- Target the introduction of vaccination to areas with high dengue transmission.
- Integrate the immunization schedule defined by the National Vaccination Council of Mexico and approved by COFEPRIS with preexisting National Health Weeks.
- Define vaccination age groups according to epidemiologic risk and producers' recommendations.
- Collect data to support national information campaigns to facilitate vaccine introduction.

Shortly after the policy recommendations produced by the MDEG were presented, a new federal administration took office. In the ensuing months, the new national public health authorities have begun to include some of those recommendations in the prevention and control programs.

## Conclusions

The introduction of new vaccines into national vaccination programs involves a series of complex political and technical decisions and requires extensive planning to secure resources needed for the successful acquisition and distribution of a vaccine. Through the analysis framework developed by the MDEG and its subsequent recommendations, Mexico has strengthened its capacity for the introduction of public health innovations such as novel vaccines and raised awareness of important factors to address. This greater capacity will facilitate the country in its aim to be an early adopter of the anticipated dengue vaccine and optimize the impact of the much-needed vaccine once it is available.

Although the work of the MDEG is considered done, it is expected that under the leadership of the FMoH, activities will continue to ensure the country's preparation for the early introduction of a new dengue vaccine. Also, it is necessary that all activities carried out before, during, and after introduction are documented so as to generate scientific evidence of the usefulness of the advanced preparedness strategy and of the vaccine introduction itself.
